# Metagenomic and Metatranscriptomic Insights into Population Diversity of *Microcystis* Blooms: Spatial and Temporal Dynamics of *mcy* Genotypes, Including a Partial Operon That Can Be Abundant and Expressed

**DOI:** 10.1128/aem.02464-21

**Published:** 2022-04-19

**Authors:** Colleen E. Yancey, Derek J. Smith, Paul A. Den Uyl, Osama G. Mohamed, Fengan Yu, Steven A. Ruberg, Justin D. Chaffin, Kelly D. Goodwin, Ashootosh Tripathi, David H. Sherman, Gregory J. Dick

**Affiliations:** a Earth and Environmental Sciences, University of Michigan, Ann Arbor, Michigan, USA; b Cooperative Institute for Great Lakes Research (CIGLR), University of Michigan, Ann Arbor, Michigan, USA; c Natural Products Discovery Core, Life Sciences Institute, University of Michigan, Ann Arbor, Michigan, USA; d Life Science Institute, University of Michigan, Ann Arbor, Michigan, USA; e Pharmacognosy Department, Faculty of Pharmacy, Cairo University, Cairo, Egypt; f National Oceanic and Atmospheric Administration (NOAA) Great Lakes Environmental Research Laboratory, Ann Arbor, Michigan, USA; g F. T. Stone Laboratory, The Ohio State University, Put-In-Bay, Ohio, USA; h Ohio Sea Grant, The Ohio State University, Put-In-Bay, Ohio, USA; i Ocean Chemistry and Ecosystems Division, Atlantic Oceanographic and Meteorological Laboratory (AOML), NOAA, Miami, Florida, USA; j Southwest Fisheries Science Center, NOAA, La Jolla, California, USA; k Department of Medicinal Chemistry, University of Michigan, Ann Arbor, Michigan, USA; Norwegian University of Life Sciences

**Keywords:** freshwater harmful algal blooms, *Microcystis*, metagenomics, *mcy* operon, Lake Erie

## Abstract

Cyanobacterial harmful algal blooms (cyanoHABs) degrade freshwater ecosystems globally. Microcystis aeruginosa often dominates cyanoHABs and produces microcystin (MC), a class of hepatotoxins that poses threats to human and animal health. Microcystin toxicity is influenced by distinct structural elements across a diversity of related molecules encoded by variant *mcy* operons. However, the composition and distribution of *mcy* operon variants in natural blooms remain poorly understood. Here, we characterized the variant composition of *mcy* genes in western Lake Erie *Microcystis* blooms from 2014 and 2018. Sampling was conducted across several spatial and temporal scales, including different bloom phases within 2014, extensive spatial coverage on the same day (2018), and frequent, autonomous sampling over a 2-week period (2018). Mapping of metagenomic and metatranscriptomic sequences to reference sequences revealed three *Microcystis mcy* genotypes: complete (all genes present [*mcyA–J*]), partial (truncated *mcyA*, complete *mcyBC*, and missing *mcyD–J*), and absent (no *mcy* genes). We also detected two different variants of *mcyB* that may influence the production of microcystin congeners. The relative abundance of these genotypes was correlated with pH and nitrate concentrations. Metatranscriptomic analysis revealed that partial operons were, at times, the most abundant genotype and expressed *in situ*, suggesting the potential biosynthesis of truncated products. Quantification of genetic divergence between genotypes suggests that the observed strains are the result of preexisting heterogeneity rather than *de novo* mutation during the sampling period. Overall, our results show that natural *Microcystis* populations contain several cooccurring *mcy* genotypes that dynamically shift in abundance spatiotemporally via strain succession and likely influence the observed diversity of the produced congeners.

**IMPORTANCE** Cyanobacteria are responsible for producing microcystins (MCs), a class of potent and structurally diverse toxins, in freshwater systems around the world. While microcystins have been studied for over 50 years, the diversity of their chemical forms and how this variation is encoded at the genetic level remain poorly understood, especially within natural populations of cyanobacterial harmful algal blooms (cyanoHABs). Here, we leverage community DNA and RNA sequences to track shifts in *mcy* genes responsible for producing microcystin, uncovering the relative abundance, expression, and variation of these genes. We studied this phenomenon in western Lake Erie, which suffers annually from cyanoHAB events, with impacts on drinking water, recreation, tourism, and commercial fishing.

## INTRODUCTION

Cyanobacterial harmful algal blooms (cyanoHABs) dominated by *Microcystis* spp. are globally distributed and reported on every continent except Antarctica (1). These toxic blooms are expected to expand in frequency and severity due to eutrophication, rising atmospheric CO_2_, and continued human-induced global warming (2–4). *Microcystis* has a complex genome that encodes many diverse, potentially harmful secondary metabolites (5–9). These compounds can contribute to an array of negative outcomes, including impacts on human and environmental health (10, 11), threats to potable water (12), and disruption of ecosystem function (2).

*Microcystis* is capable of producing the hepatotoxin microcystin (MC), a cyclic heptapeptide with over 270 reported structural variants, termed congeners (13). It is generated by a nonribosomal peptide synthetase multienzyme complex, which is encoded by a set of 10 genes in the bidirectional *mcy* operon (14, 15). This gene cluster is ancient, having originated from a common ancestor of several cyanobacteria genera, including *Microcystis*, *Dolichospermum*, and *Planktothrix* (16). The *mcy* operon also has a polyphyletic distribution within the genus *Microcystis*, providing evidence for multiple independent *mcy* gene loss events (16–18). Due to its prevalence and potential toxicity, the expression, regulation, and secretion of microcystin have been studied extensively in locations around the world (1, 6, 8, 19, 20).

**FIG 1:**
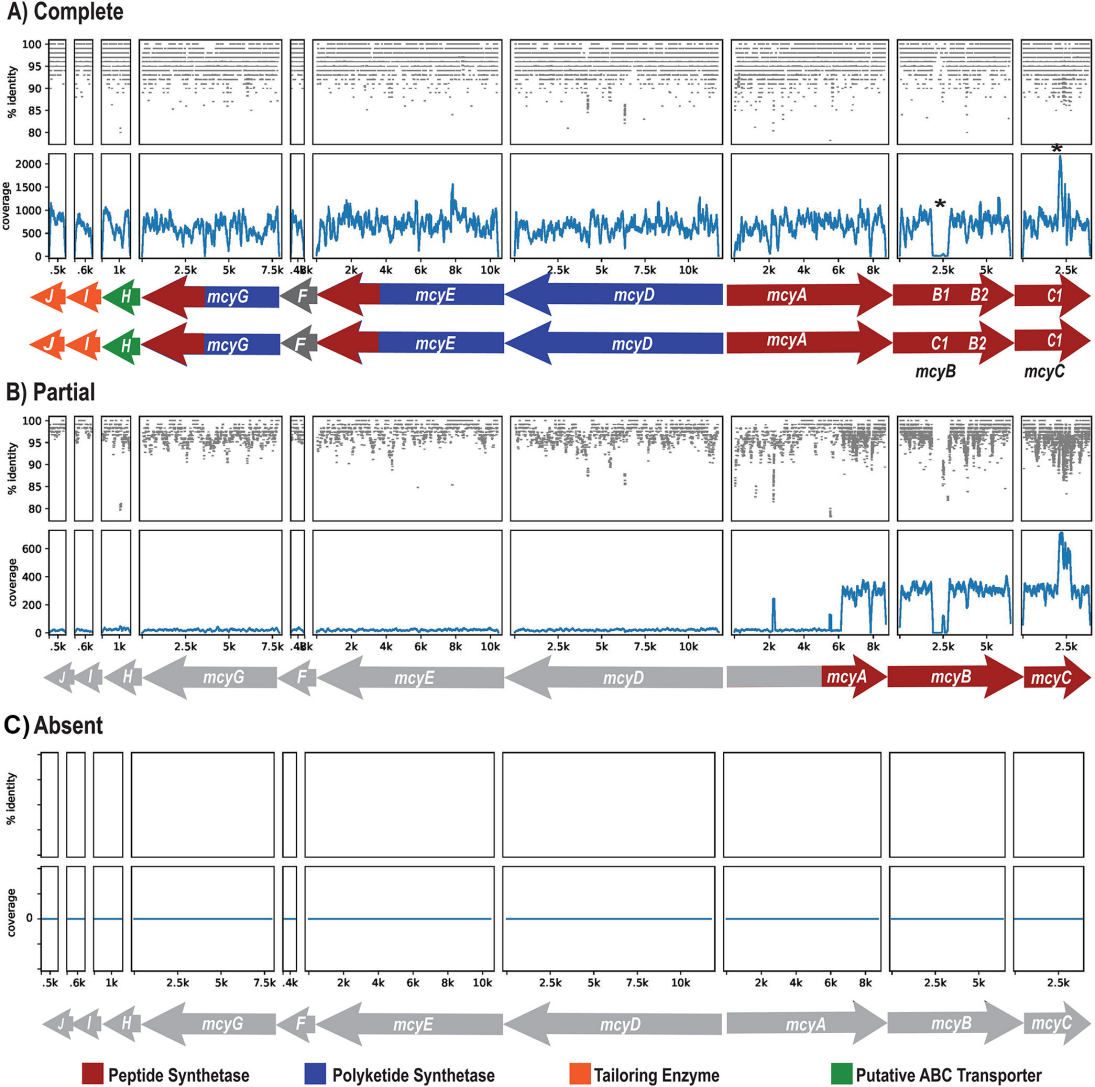
Metagenomic read mapping suggests three distinct *mcy* genotypes in western Lake Erie *Microcystis* populations at different times and locations. Genotypes were inferred by mapping metagenomic reads onto the reference *mcy* operon from Microcystis aeruginosa reference strain PCC 7806 (GenBank accession number AF183408.1). For each plot, the top panel indicates the percent identity of the read mapped to the reference operon, and the bottom panel shows coverage (number of reads mapped per position). The color of the genes indicates modular enzymes encoded by *mcy* genes. (A) Mapping results from a sample dominated by the complete genotype (WE12, 4 August). The asterisks indicate evidence of recombination in which the C1 domain replaced the B1 domain, creating a C1-like *mcyB* genotype, which is interpreted to dominate the metagenome of this sample (bottom operon). The lack of this recombination preserves the *mcyB* orientation for a B1-like *mcyB* genotype (top operon), as in the reference sequence from PCC 7806. (B) Sample dominated by the partial genotype (WE12, 23 September). (C) Absent genotype with no *mcy* genes present.

The *mcy* operon displays extensive sequence diversity (14, 21, 22), and each allele is thought to encode distinct MC congeners (21–24). However, many other factors influence the structural variations of microcystins, including the availability of amino acids in cells (25), the flexibility of amino acid adenylation domains comprising the biosynthetic enzyme complex (21), nitrogen form and availability (26), and C-to-N ratios of available nutrients (27). The genes *mcyA*, *mcyB*, and *mcyC* demonstrate the highest level of variation and a “mosaic”-like structure (21). Frequent recombination events in these genes may lead to unique gene sequences that encode distinct MC congeners, such as the replacement of the *mcyB* B1 domain with the *mcyC* C1 domain (see Fig. 1 for examples), which leads to the production of at least two MC congeners, microcystin-LR, and microcystin-RR (22).

While numerous studies have addressed how environmental variables impact congener production under controlled conditions (21, 22, 26), few have assessed the variation of the *mcy* operon structure and sequence in natural populations. Genetic research has largely focused on laboratory studies of *Microcystis* cultures aimed at understanding the *mcy* operon structure and the production of microcystin (21, 22, 24, 28). Field studies show that blooms consist of nontoxic and toxic strains (29) but have relied on PCR or consensus sequences from metagenomic assemblies (30), both of which may fail to detect important population-level variation. Mapping of metagenomic and metatranscriptomic sequence reads to reference genomes yields valuable insights into the genomic diversity and functional activity of *Microcystis* (31–33). However, these methods have not yet been widely applied to seasonal strain successions of natural *Microcystis* populations, which are an important control on bloom toxicity (18). Thus, the true environmental diversity of *Microcystis* populations remains poorly understood.

Here, we studied the diversity of the *mcy* operon in a natural *Microcystis* population in western Lake Erie, which experiences annual cyanoHABs (34). The western basin is the shallowest, warmest, and most populated of the Laurentian Great Lakes and provides drinking water to 11 million people as well as fishing and other recreation, which have substantial economic value (Western Lake Erie Basin Project, NRCS). In August 2014, Toledo, OH, experienced a drinking water crisis that left its nearly half a million residents without potable water as a result of microcystin concentrations exceeding the World Health Organization guideline of 1 microgram/L (12, 35). The 2014 western Lake Erie bloom was characterized by an early toxic phase and a late nontoxic phase that correlated with a succession of *Microcystis* strains (35, 36). This pattern of succession has been observed worldwide but is not fully understood with respect to environmental or ecological drivers (18).

We collected samples from Lake Erie during cyanoHABs in 2014 and 2018. Sampling of blooms during 2014 (35), 2018 (37), and 2018 via an autonomous 3rd-generation environmental sample processor housed within a long range-autonomous underwater vehicle (3G-ESP LRAUV) (P. A. Den Uyl, S. R. Chaganti, L. R. Thompson, R. M. Errera, C. M. Preston, W. Ussler III, C. E. Yancey, J. M. Birch, S. A. Ruberg, G. J. Doucette, G. J. Dick, C. A. Scholin, and K. D. Goodwin, submitted for publication) provided seasonal and spatial variation, high spatial resolution, and high temporal resolution, respectively. The 2014 cyanoHAB data provided insights into spatial and temporal shifts from weekly samples collected from three stations from July to October (35). The 2018 “HABs Grab” data set achieved higher spatial resolution via 25 samples collected from various points in the lake on the same day (37). The 2018 3G-ESP captured high temporal resolution through autonomous sample collection over a 2-week period (Den Uyl et al., submitted). Analysis of the *mcy* genes and strain diversity in these data sets was used to assess novel genotypes and their relative frequencies as well as high genomic variation across time and space within the western basin and determine the relationships between genotypes and environmental variables to determine patterns of ecological strain succession.

## RESULTS

### *mcy* operon coverage and genotypes.

Mapping of metagenomic sequence reads to the *mcy* operon from the reference genome Microcystis aeruginosa PCC 7806 revealed three major *mcy* genotypes in western Lake Erie samples. First, relatively even coverage across the operon indicated dominance by a complete genotype with all 10 *mcy* genes present (Fig. 1A). Second, a partial *mcy* genotype was inferred through the detectable coverage of half of the *mcyA* gene and the full *mcyB* and *mcyC* genes but essentially no coverage of other *mcy* genes (Fig. 1B). A third, “absent” genotype was inferred through no coverage of *mcy* genes despite the presence of *Microcystis* in a sample, indicating *Microcystis* genotypes in which no *mcy* genes were present (Fig. 1C). Where *mcyB* and *mcyC* genes were present, mapping resulted in a lack of coverage at the location of the B1 domain (∼2.5 kb of *mcyB*) and approximately double coverage of the C1 domain (∼2.5 kb of *mcyC*) (Fig. 1A). This is indicative of a recombination event in which the *mcyB* B1 domain is replaced by the *mcyC* C1 domain as described previously by Mikalsen et al. (22).

**FIG 2:**
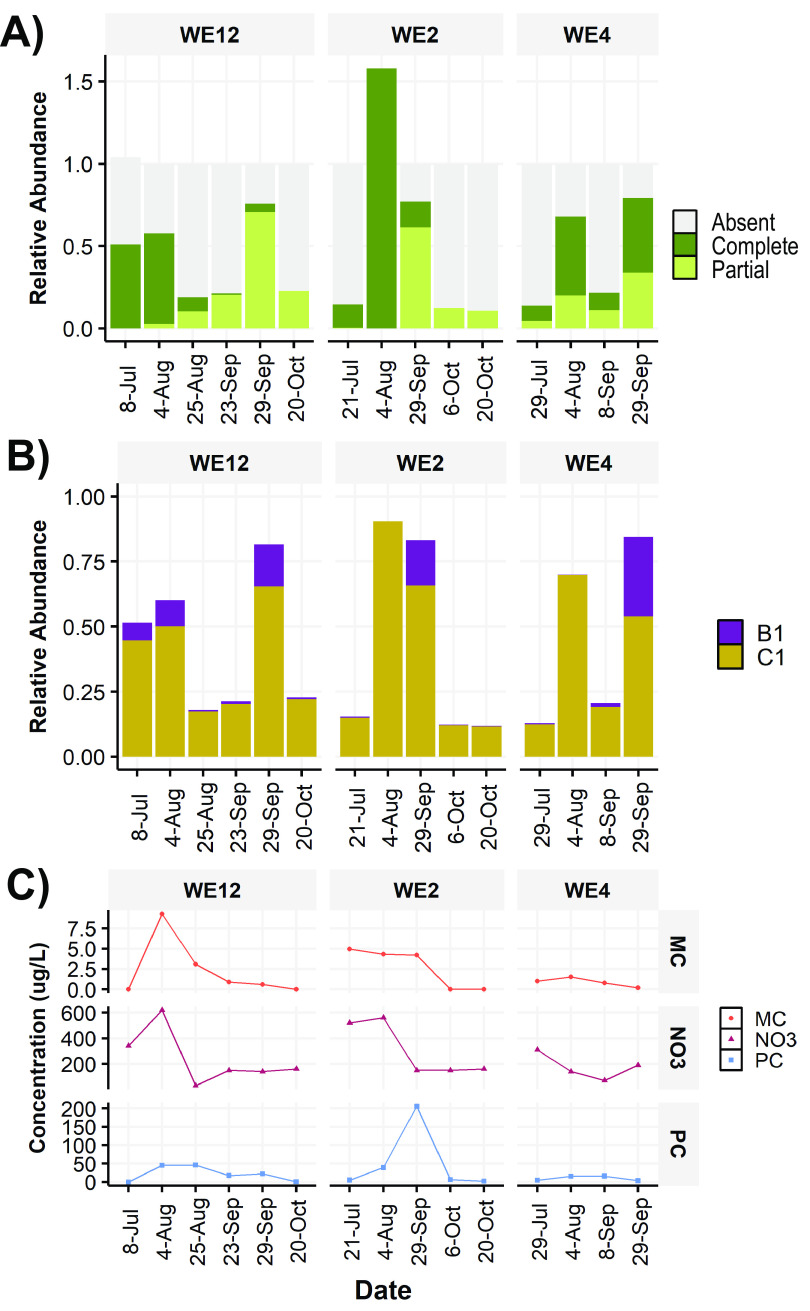
Relative abundances of *Microcystis mcy* genotypes from the 2014 cyanoHAB by station and date. Relative abundances of genotypes were calculated by taking the ratio of the coverage of *mcy* genes to the coverage of the V4 16S rRNA gene. (A) Proportion of the *Microcystis* population that contained a complete, partial, or absent *mcy* operon by sample. Values above 1 indicate higher coverage of *mcy* genes than the 16S rRNA genes (see Materials and Methods). (B) Proportion of the *Microcystis* population that contains either the *mcyB* C1-like or B1-like variant. (C) Concentrations of particulate microcystins (MCs), nitrate (NO_3_^−^), and phycocyanin (PC) per sample (micrograms per liter).

The relative abundances of the *Microcystis* populations that contained a complete, partial, or absent *mcy* genotype from each station were estimated by comparison of *mcy* gene coverage with *Microcystis* 16S rRNA gene coverage (see Materials and Methods). The estimated relative abundance of each *mcy* genotype in 2014 cyanoHAB samples shifted with location and bloom conditions (Fig. 2). Relative abundances are reported in unitless ratios from 0-1 with 0 indicating the absence of a gene or genotype, and 1 indicating a 1 to 1 ratio of the mcy gene to the 16s rRNA gene. At nearshore station WE12, complete genotypes accounted for approximately half (0.51 to 0.54) of the *Microcystis* population during the toxic, nitrate-replete bloom in July and early August, but absent and partial *mcy* genotypes dominated from late August through October, after microcystin and nitrate concentrations declined (Fig. 2C). At the other nearshore station, WE2, a similar transition from complete to absent and partial *mcy* genotypes was observed. During the peak phase of the bloom at WE2 (4 August), the complete genotype coverage ratio was 1.58, indicating that *mcy* genes were more abundant than 16S rRNA genes at this time. At the offshore station (WE4), *mcy* genotypes were more mixed (Fig. 2A). The *mcyC1* recombination variant was the most abundant (e.g., replacing the *mcyB1* genotype) at all times and stations (Fig. 2B).

**FIG 3:**
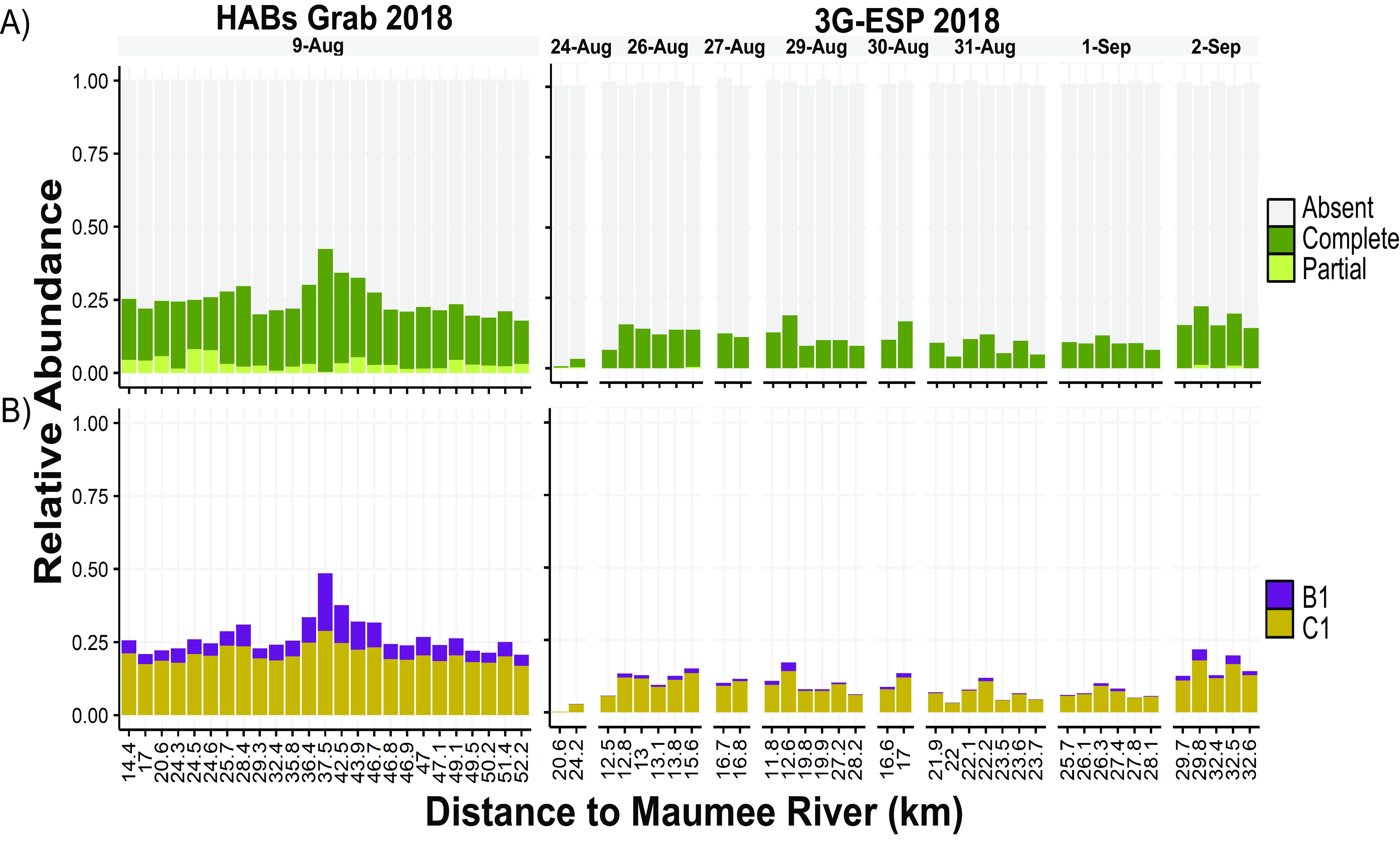
Relative abundances of *mcy* genotypes for *Microcystis* populations sampled during the 2018 HABs Grab and 2018 ESP monitoring efforts. Relative abundances were calculated by taking the ratio of the coverage of *mcy* genes to the coverage of the V4 16S rRNA gene. The *x* axis is the distance from the Maumee River (kilometers) from which the sample was collected. (A) Proportion of the population containing a complete, partial, or absent *mcy* operon. (B) Proportion of the population containing the C1-like or B1-like *mcyB* variant for both the 2018 HABs Grab and 3G-ESP sampling efforts.

We next calculated the relative abundances of *mcy* genotypes in the 2018 HABs Grab (37) and the 2018 3G-ESP LRAUV (Den Uyl et al., submitted) data sets to assess dynamics over narrow spatial and temporal scales, respectively, compared to the 2014 data set. In every 2018 sample, the most abundant *mcy* genotype was the absent genotype (Fig. 3A). Among *mcyB* genes, the C1 variant was most abundant (Fig. 3B). Samples from the HABs Grab effort were compared based on their distances from the mouth of the Maumee River, the major source of nutrient runoff for western Lake Erie (38). The relative abundance of the complete *mcy* operon genotype was highest (0.42) in samples 37 to 47 km from the mouth of the Maumee River, peaking at 37.5 km (Fig. 3).

3G-ESP data from 2018 also showed low relative abundances of complete and partial *mcy* genotypes (<0.20) throughout the 2-week sampling period. While strong spatial or temporal trends were not observed during this period, variability was observed across small spatial scales (<0.5 km) (Fig. 3).

### *mcy* genotypes and environmental variable correlations.

The relative abundances of *mcy* genotypes in the 2014 *Microcystis* population showed significant correlations with several environmental variables (Table 1; see also Table S1 in the supplemental material). The relative abundance of the complete *mcy* operon had significant positive correlations with pH (Pearson’s *R* = 0.51 [*P* = 0.05]) and nitrate concentrations (Pearson’s *R* = 0.62 [*P* = 0.013]) and a nonsignificant relationship with ammonium concentrations. The relative abundance of the partial *mcy* genotype was significantly and negatively correlated with both nitrate and ammonium concentrations (Pearson’s *R* = −0.53 [*P* = 0.044] and Pearson’s *R* = −0.52 [*P* = 0.048], respectively). The relative abundance of the absent genotype was significantly and negatively correlated with pH (Pearson’s *R* = −0.54 [*P* = 0.037]) and nonsignificantly and positively correlated with ammonium concentrations (Pearson’s *R* = 0.40 [*P* = 0.14]) (Table 1). The relative abundance of the C1/B1 *mcyB* variant showed a positive, significant correlation with dissolved organic carbon (DOC) (Pearson’s *R* = 0.580 [*P* = 0.022]) (Table S2).

**TABLE 1:** Pearson’s correlations generated for the relative proportion of the *Microcystis* population that had a complete, partial, or absent *mcy* operon as well as the ratio of C1/B1 variants against the environmental variables pH, nitrate concentration, and ammonium concentration (*n* = 15)

*mcy* genotype category	pH		Nitrate concn (mg/L)		Ammonium concn (μg/L)	
	*R*	*P* value	*R*	*P* value	*R*	*P* value
Complete	0.513	0.05	0.621	0.013	−0.082	0.77
Partial	−0.0318	0.91	−0.525	0.044	−0.52	0.048
Absent	−0.541	0.037	−0.359	0.19	0.403	0.14

### *Microcystis* strain diversity.

**FIG 4:**
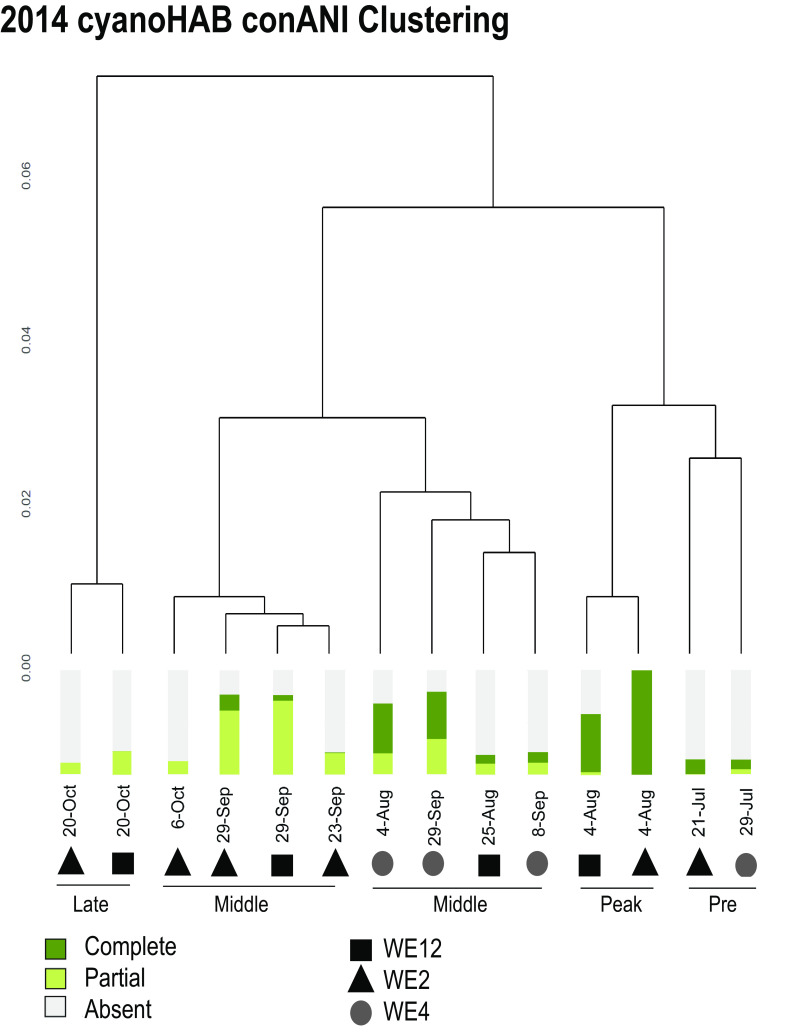
Hierarchical clustering of 2014 metagenomic samples based on pairwise conANI comparisons. Clustering indicates that the *Microcystis* strain composition is more similar in samples from the same bloom phase than in those from different phases that tend to have the same dominant genotype. The bloom phase described as “middle” in the illustration above refers to samples collected in August and September where the *Microcystis* biomass and microcystin concentration are still detectable yet past peak concentrations.

To determine if *mcy* genotypes were a result of the presence of multiple strains or recent gene transfer events, we calculated genome-wide strain variation of *Microcystis* in the 2014 cyanoHAB metagenomes based on single nucleotide variants (SNVs) (39). Consensus average nucleotide identity (conANI), a measure of SNVs between samples (see Materials and Methods), was calculated for *Microcystis* in all samples. Hierarchical clustering of conANI values based on pairwise comparisons showed that samples clustered based on the bloom phase (Fig. 4). ConANI values between samples ranged from 97.3014% to 99.7936% (Table S2). These ranges provide strong evidence that these samples consist of multiple strains as a value of 99.999% has been shown to be a sufficiently stringent cutoff for ANI measurements in identifying identical strains within metagenomic data sets (39). There was a clear distinction in sample clustering between the peak phase of the bloom, where the populations were predominantly composed of strains that contained the complete *mcy* operon, and the mid- to late phases, where the partial and absent genotypes were more prevalent. These results suggested that the *Microcystis* strains present during different bloom phases were distinct in terms of their genome-wide nucleotide similarity and that changes in *mcy* genotype relative abundances cannot be explained by recent gene transfer events.

ConANI comparisons measure the difference in SNVs present between two samples and, thus, represent a minimum estimate of the number of mutations that distinguish the *Microcystis* strains in two samples. To evaluate whether the observed SNV differences between samples could feasibly arise from mutations formed during the course of a single bloom season, we used upper and lower limits of bacterial mutation rates (40) to estimate the divergence time required to produce the observed conANI values. The results indicated this to be unlikely because thousands to millions of years would be required to account for the observed genome-wide differences, and hundreds to thousands of years would be required to account for nucleotide differences in *mcy* genes between samples containing complete and partial genotypes (Table 2).

**TABLE 2:** Estimations of times of divergence for *Microcystis* strains observed in the 2014 cyanoHAB^*a*^

Sequence	Mutation rate (no. of substitutions/site/yr)	Time of divergence (yrs)
Whole genome	1.00E−04	1.06E+06–1.39E+07
	1.00E−07	1.06E+09–1.39E+10

*mcyB*	1.00E−04	3.53E+02–1.24E+04
	1.00E−07	3.53E+05–1.24E+07

*mcyC*	1.00E−04	3.00E+02–1.24E+04
	1.00E−07	3.00E+02–1.24E+07

a Mutation rates included in this table represent the upper and lower limits of mutation rates found in several bacterial genomes based on an analysis completed by Gibson and Eyre-Walker (40). Times of divergence were calculated for the samples that had the lowest and highest conANI scores for whole genomes and the *mcyB* and *mcyC* genes compared pairwise.

### *mcy* gene expression.

**FIG 5:**
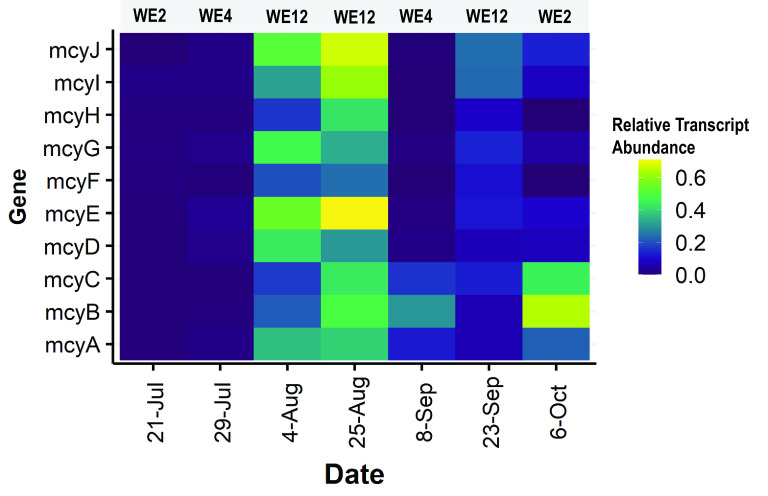
Heatmaps showing the relative abundances of metatranscriptomic reads mapped to the *mcy* operon. Relative abundance is determined by calculating the coverage of the gene of interest (number of transcript reads mapped divided by gene length) divided by the coverage of transcripts mapped to the entire genome divided by genome length.

Metatranscriptomic data were analyzed to track the relative abundances of *mcy* gene transcripts over time and space in samples from the 2014 bloom. The transcript relative abundance of the complete *mcy* operon was highest at nearshore station WE12 on all three dates sampled (Fig. 5). The transcript relative abundance was low (near detection limits) during the early phases of the bloom, as seen at nearshore station WE2 on 21 July and offshore station WE4 on 29 July. When the partial *mcy* operon was more prevalent in the population than the complete operon genotype (Fig. 2), the partial genes were expressed (Fig. 5), as observed at station WE2 on 6 October and at station WE4 on 8 September.

## DISCUSSION

We used metagenomic and metatranscriptomic data to reveal the dynamics of *Microcystis* strains across a variety of spatial and seasonal scales in 2014 and 2018 blooms in western Lake Erie. The 2014 bloom presented the opportunity to investigate a seasonal succession of *Microcystis* strains alongside changing environmental conditions (35). An early toxic phase of the bloom (i.e., high concentrations of MCs) that coincided with high nitrate (nitrate-N > 400 μg/L) conditions (41) gave way to a nontoxic phase of the bloom with nitrate-depleted conditions (nitrate-N < 200 μg/L later in the season) (Fig. 2 and Table 1). In addition to the widely known toxigenic and nontoxigenic *Microcystis* genotypes (all *mcy* genes present and absent, respectively), a genotype with a partial set of genes (*mcyA–C*) was found to be abundant and expressed in the nontoxic phase of the bloom.

The significant correlations between *mcy* genotype relative abundance estimates and concentrations of ammonium and nitrate and pH (Fig. 2 and Table 1) suggest that nitrogen availability and the photosynthetic rate of the bloom, which drives extreme changes in pH (33), may influence the competitive fitness of each genotype and thus shape the *Microcystis* strain composition in the blooms. *Microcystis* also showed microdiversity across the whole genome. Nucleotide similarity between samples was clustered by bloom phase (Fig. 4), indicating that discrete subpopulations (7) may be ecologically distinct as well. Based on known mutation rates, genetic divergence between subpopulations was estimated to require at least thousands of years, indicating that the observed succession involved primarily ecological selection rather than the evolution of genotypes within the season.

Our approach for estimating the relative abundances of genotypes through coverage ratios of metagenomic sequence reads that mapped to the *mcy* gene and the 16S rRNA gene was subject to several uncertainties. First, whereas it is commonly assumed that *Microcystis* genomes contain one copy of the *mcy* and rRNA operons (42, 43), all nine closed genomes of *Microcystis* that were publicly available have two copies of the 16S rRNA gene and one copy of the *mcy* operon (44). The gene copy numbers of *mcy* and 16S rRNA genes in native populations are uncertain (45) and may vary from strain to strain. Variation in coverage and, thus, our estimates of relative abundance could be affected by both the *mcy* gene copy number per genome as well as the relative abundance of cells with the various *mcy* genotypes. Second, rapid genome replication can result in gene dose effects wherein genes near the origin of replication are present in higher copy numbers than those at the terminus (46–48). The presence of *mcy* genes near the origin of replication or multiple copies of *mcy* genes per genome may explain our reported coverage ratio value of 1.58 at WE2 on 4 August 2014 (Fig. 2), but the available data cannot determine whether these factors were actually at play in these western Lake Erie populations. Third, the observed gene counts could be affected by nonspecific matches to V4 16S rRNA and *mcy* genes from other cyanobacteria (e.g., *Dolichospermum*) or homologous biosynthetic genes. However, we used stringent thresholds and aligned all mapped *mcy* and 16S rRNA gene sequence reads against a custom universal database and the Silva SSU DB v.138 database, respectively, to confirm that there was no significant spurious mapping. Despite these limitations, the relative abundances measured here track major shifts in genotypes across spatiotemporal scales, and our values are consistent with previous findings in western Lake Erie blooms (29). While the read-level analysis conducted here has long been used to study the population-level diversity of microbial communities (49, 50), it has not been widely applied to studies of cyanobacterial blooms.

The results from this study reveal that natural *Microcystis* populations consist of multiple *mcy* genotypes, some of which have been described previously from studies of pure cultures, such as the complete and absent genotypes (21, 22, 51). In this study, the *Microcystis* population was dominated at times by genotypes that lack *mcy* genes altogether (throughout 2018) (Fig. 3) or by a genotype in which only the *mcyA–C* genes were present (late September in 2014) (Fig. 2). This partial *mcy* genotype is present in just 1 of 159 publicly available *Microcystis* genomes derived from culture (PCC 9717; NCBI assembly ASM31216v1), which was isolated from a water dam in Rochereau, La Vendée, France. To our knowledge, this partial genotype has been briefly mentioned only once in the literature, by Pearson et al. (6), who identified it in a PCC 9717 culture. Interestingly, we detected the partial genotype across multiple locations and dates spanning 4 years (Fig. 2 and 3), indicating that it is a persistent member of the *Microcystis* bloom community in Lake Erie. The partial genotype was the most abundant during the secondary bloom phase of 2014 where conditions were nutrient depleted (29, 52). This persistence and occasional dominance, together with the active transcription of genes from this partial operon (Fig. 5), suggest that this strain is ecologically successful and that the partial *mcy* operon may be functional. The significant negative correlation of the partial genotype ratio estimate with both nitrate and ammonium concentrations (Table 1) suggests that it is adapted to conditions of low N. Experiments on pure cultures will be helpful in addressing open questions on the ecology, biosynthetic potential, and toxigenicity of this partial *mcy* genotype.

The complete *mcy* genotype often dominated in the early stages of the bloom under nitrate-replete conditions (Fig. 2A and C). Only the complete *mcy* genotype coverage ratio was positively correlated with nitrate concentrations (Table 1). This observation was consistent with microcystin-producing strains having a higher demand for nitrogen (41, 52, 53) due to the high N content of microcystin metabolites as predicted by nutrient stoichiometry theory (54, 55). Similarly, only the complete *mcy* genotype was positively correlated with pH (Table 1). This finding suggests that MC-producing strains were associated with increased photosynthetic rates and/or high pH relative to non-MC-producing strains. Increasing photosynthetic rates greatly increase the pH and serve as a good proxy for bloom activity (56, 57). This agrees with previous results showing that faster-growing strains tend to be MC producers (54) and that shifts in *Microcystis* genotypes correlated strongly with pH, with potential links to carbon acquisition and concentrating mechanisms (33). Lower pH generally indicates cyanoHAB communities dominated by slow growth and maintenance, which may be a specialty of nontoxigenic strains (54, 57).

Our results show that several different *mcy* variants, reflecting the mosaic structure of the *mcy* operon identified in pure cultures, co-occur in natural environments. The study by Mikalsen et al. (22) demonstrated that the sequence of the *mcyB1* domain (e.g., either a B1-like or a C1-like genotype) influences the MC congener produced within cultured isolates. *Microcystis* strains with the B1-like genotype produced LR isoforms, while those with C1-like genotypes produced both LR and RR isoforms (22). We detected both the B1 and C1 genotypes in western Lake Erie populations, providing genetic context for the production of MC-LR and -RR, two of the most common congeners in this system (37, 58). The LR- and RR-producing C1 variant was the most abundant form of the *mcyB* gene in western Lake Erie during both 2014 (Fig. 2) and 2018 (Fig. 3) as well as 2006 (59), showing that these genotypes are present interannually. While our study focused on *mcyB* variants, it is likely that other genotypes are present in these populations, especially within the hyperdynamic *mcyABC* region.

Given the dynamic nature of *Microcystis* genomes (5) and evidence of genome rearrangement in response to environmental conditions (31), one competing hypothesis explaining seasonal trends in genotypic diversity is active horizontal gene transfer (HGT) or loss of *mcy* genes during the season. However, the distinct clustering of samples by bloom phase based on genome-wide conANI scores (Fig. 4) suggested that the seasonal dynamics of *mcy* genotypes were due to shifts in the abundances of *Microcystis* strains rather than ongoing evolution at the time scale of this study. If the differential coverage ratios of *mcy* genotypes were due to active HGT and/or gene loss, the same strains would be present at all times, and the conANI would be at least 99.999% (39); in contrast, we found that the values ranged from 97.3014% to 99.7936% (see Table S2 in the supplemental material). We conclude that the samples contained distinct strains that shifted in abundance over the course of the bloom and diverged on the order of thousands of to several million years based on whole-genome analysis and a wide range of mutation accumulation rates (Table 2). While conANI results varied across genes, as expected due to different evolutionary histories and forces (6, 21–23), even the shortest estimated divergence times were on the scale of hundreds to thousands of years. Rather than HGT, the results from this study suggested that specific genotypes were favored during the bloom according to shifting niche space, with an emphasis on nitrogen form and availability as well as pH (Table 1).

The co-occurrence of various *mcy* genotypes in natural cyanobacterial bloom communities highlights that genotypes commonly observed in culture (21, 22, 51) as well as novel genotypes that have scarcely been mentioned in the literature (6) are common in natural populations. Our results show that this genotype diversity shifts dynamically in time and space and suggest that it is likely responsible in part, along with the direct influence of environmental conditions, for the diversity of structural variants of MC observed in nature. The presence of an abundant, transcriptionally active, partial *mcy* operon of unknown biosynthetic potential merits further investigation. Its presence further highlights the potential pitfalls of using single *mcy* genes to assess MC-producing strains, as *mcyA–C* will not distinguish partial and complete genotypes, and the use of *mcyD* or *mcyE* will not capture the presence of the partial genotype. For example, the Phytotoxigene (Akron, OH, USA) quantitative PCR (qPCR) screening kit, which is commonly used to detect MC-producing potential, detects only the *mcyE* gene. Such an approach would not detect the partial operon identified in this study. Overall, this study shows the utility of shotgun omics approaches for resolving known and novel *mcy* genotypes that can dominate natural blooms with the potential for new phenotypic outcomes of concern to human and animal health.

## MATERIALS AND METHODS

### Sample collection.

**FIG 6:**
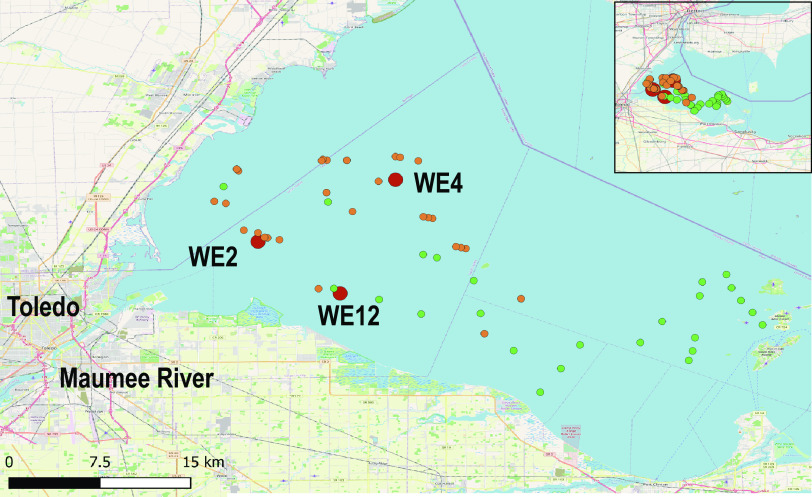
Map of western Lake Erie HAB long-term monitoring stations, 2018 HABs Grab sample locations, and 2018 3G-ESP sample collection points. Western Lake Erie long-term monitoring stations are denoted with large red circles, 2018 HABs Grab samples are green circles, and 3G-ESP sample locations are orange circles. (The Open Street Map [OSM] [https://wiki.osmfoundation.org/wiki/Main_Page] was used as the basis for the map.)

The 2014 cyanoHAB samples were collected from NOAA Great Lakes Environmental Research Laboratory (GLERL) sampling stations WE2, WE4, and WE12 (Fig. 6) throughout the western basin of Lake Erie from mid-June through late October 2014 (60). WE2 is close to the inlet for the Maumee River (41°45.743′N, 83°19.874′W), WE4 is considered an offshore site closer to the center of the basin (41°49.595′N, 83°11.698′W), and WE12 is proximal to the Toledo drinking water inlet (41°42.535′N, 83°14.989′W). Given the propensity of *Microcystis* to migrate vertically through the water column and occur at varying depths, 20-L integrated-depth samples were collected from each station for biological and chemical analyses. Integrated depths were defined as the surface of the water to 1 m above the lake floor. While samples were collected, measurements of pH and water temperature were collected as well. At the time of collection, GLERL also collected samples to measure various environmental variables, including nutrient and pigment concentrations. In order to capture *Microcystis* aggregates, 2 L of integrated-depth-collected water was filtered through a 100-μm polycarbonate mesh filter. The biomass on the filter was then collected and filtered onto a 0.22-μm filter. The biomass on the 0.22-μm filter was preserved in 1 mL of RNAlater (Invitrogen, Ambion) and placed on ice. Upon arrival at the laboratory, samples were stored in a −80°C freezer until DNA and RNA extractions could be completed.

We also analyzed shotgun data from samples collected during the western Lake Erie 2018 HABs Grab and 2018 ESP sampling campaigns (Fig. 6). These studies have been outlined elsewhere (37; Den Uyl et al., submitted). Briefly, the 2018 HABs Grab aimed at achieving high spatial sampling on a single day. Samples (*n* = 100) were collected on 9 August 2018, with a wide range of accompanying environmental data. Integrated-depth water samples were collected using a 2-m-long tube sampler for an integrated sample from the surface to 2 m deep, and the sample water from the tube sampler was deposited into a clean 20-L bucket. Two liters of water was poured into a polyethylene terephthalate glycol (PETG) bottle per station and stored in dark coolers until processing was completed at the laboratory later that day. EXO2 sonde (YSI Inc., Yellow Springs, OH, USA) was used to measure sample parameters, including temperature, pH, specific conductivity, turbidity, and chlorophyll and phycocyanin fluorescence, within 60 s of collection using an EXO calibration cup. A subset of samples (*n* = 25) was selected for DNA extraction off 25-mm 1.2-μm-pore-size filters (Versapor acrylic 297 copolymer, part number 66393; Pall, Port Washington, NY, USA).

The 2018 3rd-generation environmental sampling processer (3G-ESP) sampling effort was performed over a 2-week period spanning 24 August to 2 September 2018, with an autonomous sampling vehicle. The 3G-ESP was integrated into the payload of a Tethys-class long-range autonomous underwater vehicle (LRAUV) (61, 62). Water samples were collected via an intake tube, pumped through an internal cartridge within the 3G-ESP with water filtered onto stacked 5-μm and 0.22-μm filters, preserved with RNAlater (Invitrogen, Ambion) on the vehicle, and archived for retrieval once the vehicle was returned to the laboratory. The 5-μm filters were used for subsequent metagenomic analysis. Samples were collected 2 to 6 m below the surface to ensure the complete submersion of the vehicle, and water was continuously collected until each filter was clogged (23 to 100 mL). Filter clogging was determined by a pressure sensor setting within the instrument.

### Sample processing and sequencing.

The 2014 bloom DNA extractions were completed using DNeasy blood and tissue extraction kits with QIAshredder columns according to the protocol provided by the manufacturer (Qiagen, Hilden, Germany). The extracted DNA concentration was determined using the Quant-iT PicoGreen double-stranded (dsDNA) assay kit. RNA extractions were completed using the Qiagen (Hilden, Germany) RNeasy kit according to the manufacturer’s protocol. Shotgun DNA and RNA sequencing was completed at the University of Michigan DNA Sequencing Core. Sequencing was completed on the Illumina HiSeq platform (2000 PE 100; Illumina Inc., San Diego, CA, USA).

For the 25 samples from the 2018 HABs Grab sampling effort DNA was extracted, quantified, and sequenced in a prior study as described previously (37).

The 2018 ESP data samples were processed and sequenced according to the following protocol. Filters were recovered from the archived cartridges upon the completion of the 2-week sampling effort and stored at −80°C until further processing was completed. To complete DNA sequencing, filters were removed from the freezer and subjected to the Qiagen DNeasy blood and tissue extraction protocol using QIAshredder columns according to the manufacturer’s directions (Qiagen, Hilden, Germany). The purity and quantity of DNA were determined using a NanoDrop Lite spectrophotometer (Thermo Scientific, Waltham, MA, USA). DNA samples were submitted to the University of Michigan Advanced Genomics Core for genomic library construction and sequencing. Sequencing was completed on an Illumina NovaSeq 6000 system equipped with an S4 flow cell with 300 cycles (150-bp paired ends).

### *mcy* operon analysis.

Trimming, adaptor removal, and quality check (QC) of raw reads were completed using a suite of tools from the IMG-JGI BBTools package (63). Reads that satisfied quality check parameters were saved and used for subsequent analysis. QC forward and reverse reads were mapped or aligned to sequences using BLAST v.2.8.1. (64).

Given that all *Microcystis* cells contain a 16S rRNA gene but not all *Microcystis* cells have *mcy* genes, we used the ratio of the coverages of these genes to estimate the relative abundance of *Microcystis* cells that have *mcy* genes out of the total *Microcystis* population. The coverage ratio of *mcy* to V4 16S rRNA gene regions for *Microcystis* was calculated by dividing the number of reads mapped to *mcy* genes by the number of reads mapped to the *Microcystis* 16S rRNA gene V4 variable region, normalized by gene lengths (lengths are recorded in Table S4 in the supplemental material). Mapping databases for both *mcy* and V4 16S rRNA genes can be accessed at https://github.com/ceyancey/mcyGenotypes-databases. Reads that mapped to all 10 *mcy* genes (Fig. 1) and 16S rRNA V4 for *Microcystis* were quantified and normalized by gene length, as shown by the following equation:

Coverage ratio (Microcystis mcy/16S) = number of reads mapped to mcy gene X/length of mcy gene X (bp)number of reads mapped to Microcystis 16S V4/length of Microcystis 16S V4 (bp)

The 16S mapping database was constructed using the V4 regions available for the phylum *Cyanobacteria* using the criteria of 95% identity and 80% alignment length, with sequences accessed from the SILVA 138.1 database in February 2021 (65). The use of these cutoffs and the V4 region was shown to be sensitive, specific, and sufficient for 16S rRNA gene mapping as the V4 variable region is more specific to the target organism than the entire 16S rRNA gene sequence. Mapping analysis also demonstrated that these cutoffs ensure that only *Microcystis* reads mapped to the V4 region. To identify *mcy* genes in the shotgun metagenomic data, all *mcy* gene sequences found in the JGI IMG/MER (66) database (accessed February 2021) were used, as were sequences from closely related cyanobacteria to maintain competitive mapping. Reads were kept for downstream analysis if they were at least 95% identical to the reference gene, 80% in alignment length, and the singular top hit per read. These cutoff parameters, database metrics, and mapping tools were tested exhaustively to ensure specificity and sensitivity by cross-checking matches to the full SILVA database and a universal database (https://github.com/TealFurnholm/Universal_Microbiomics_Alignment_Database), and extensive analysis was completed on the mapping method to ensure accuracy and remove any erroneous, nonspecific read mapping (data not shown).

Coverage ratios were then used to estimate the relative abundances of complete, partial, or absent *mcy* operon genotypes. The complete, partial, and absent genotypes were initially identified by viewing the metagenomic coverage of the PCC 7806 *mcy* operon (GenBank accession number AF183408.1) (see Fig. 1 for examples). First, the relative proportions of the complete operon were determined by averaging the coverage ratios (*mcy* gene/*Microcystis* V4 16S rRNA) of the population that contained the *mcyD–J* genes. Next, the relative abundance of the partial operon genotype was estimated by subtracting the average *mcy* gene/V4 16S rRNA coverage ratio of the *Microcystis* population that had the complete operon from the average ratio of the population that contained the *mcyA–C* genes. Finally, the relative abundance of the population that did not contain any *mcy* genes, i.e., the absent genotype, was estimated by subtracting the coverage ratio of the population that contained the complete genotype plus the coverage ratio that contained the partial genotype from 1 (representing the entire population).

The relative abundances of *mcyB* B1 and C1 variants were quantified as follows. The PCC 7806 *mcy* operon contains an *mcyB* region with a B1 variant; thus, reads that mapped to the B1 domain specifically were counted and used to calculate the proportion of the population that contained the B1 variant using the equation listed above. The *mcyB1* domain region is between bp coordinates 2156 and 3111 as described previously by Mikalsen et al. (22). The estimated relative abundance of *Microcystis* that contained the C1 variant was then determined by subtracting the relative abundance of the B1 variant from the total portion that contained the *mcyB* gene.

### *mcy* genotypes and environmental variable correlation analysis.

To assess the relationships between the *mcy* genotypes and environmental conditions, a series of simple linear regressions were generated for the 2014 cyanoHAB data. The 2018 HABs Grab and ESP data were excluded from this data set because these data sets did not pair with similar environmental condition measurements. Pearson’s correlations were generated for the estimates of the population that contained the complete, partial, or absent *mcy* genotype as well as the *mcyB* B1 or C1 variant against several environmental variables, including pH, the mass of nitrogen as measured through nitrate concentration (micrograms per liter), and ammonium concentration (micrograms per liter) (*n* = 15). For each correlation, Pearson’s correlation coefficient, *r*, was generated, as was a *P* value, to determine the strength of the correlation (α = 0.05) respectively.

### *mcy* operon expression analysis.

In order to analyze *mcy* gene expression, metatranscriptomic reads were mapped to the reference *mcy* operon database used for metagenomic analysis for a select number of samples with paired metatranscriptomes from the 2014 cyanoHAB. BLAST was used with parameters identical to those used for metagenomic mapping. In order to normalize gene expression, metatranscriptomic reads were also mapped to the entire reference genome Microcystis aeruginosa PCC 7806SL (GenBank accession number CP020771.1; GI:1181755937). In order to achieve competitive genome mapping, this database contained complete genomes from *Anabaena* sp. strain 90 (accession number GCA_000312705.1), *Cyanobium* sp. strain NIES-981 (accession number GCA_900088535.1), and *Pseudanabaena* sp. strain PCC 7367 (accession number GCA_000317065.1), which are other common cyanobacterial taxa found in western Lake Erie cyanoHABs. The following equation was used to normalize metatranscriptomic reads per gene/entire *Microcystis* genome expression (67):

$$\begin{array}{l} \text{Expression (relative transcript abundance)}\\ =\frac{\text{number of reads mapped per gene }X/\text{length of gene }X\text{ (bp)}}{\text{total number of reads mapped to PCC }7806\text{SL genome/length of PCC}7806\text{ SL genome (bp)}} \end{array}$$

### Strain variation detection.

In order to determine if samples consisted of identical or distinct strains of *Microcystis*, inStrain software (39) was implemented. Briefly, metagenomic reads were mapped to a database that contained reference genomes of commonly occurring cyanobacteria in western Lake Erie, Microcystis aeruginosa PCC 7806SL (GenBank accession number CP020771.1; GI:1181755937), *Anabaena* sp. 90 (accession number GCA_000312705.1), *Cyanobium* sp. NIES-981 (accession number GCA_900088535.1), and *Pseudanabaena* sp. PCC 7367 (accession number GCA_000317065.1), using bbmap (63) with a 90% identity threshold. The generated bam files were profiled using default parameters in inStrain, and profiles were compared using the compare function, with default settings. Measurements of average nucleotide identity (ANI), specifically consensus, or conANI, were used to determine whether samples contained identical or distinct strains of *Microcystis* as discussed previously by Olm et al. (39). Olm et al. describe a cutoff of 99.999% as the threshold for identical strains as being a stringent and sufficient metric for metagenomic data and that samples that share the same strain but had undergone a recent transmission event would still have an ANI score of at least 99.999% (39). Therefore, we used this cutoff to conclude if our samples were comprised of identical or different strains. Using the conANI metric, a similarity matrix of samples was rendered, and a dendrogram was generated to cluster populations by samples. The same method was applied to determine pairwise conANI values for the *mcyB* and *mcyC* genes as well. The sample collected at WE12 on 8 July was omitted as it did not pass the coverage thresholds described by Olm et al. for comparison.

To estimate the time of divergence between samples for whole-genome comparisons and *mcy* gene comparisons, a few basic calculations were completed. Mutation rates for the 16S rRNA gene of cyanobacteria have been calculated to be 5.7 × 10^−7^ substitutions/site/year for a *Microcoleus* operational taxonomic unit (OTU) and 2.3 × 10^−7^ substitutions/site/year for a *Geitlerninema* OTU (68). An analysis completed by Gibson and Eyre-Walker demonstrated that the majority of mutation rates analyzed for 34 bacterial species fell in the range of 1 × 10^−7^ to 1 × 10^−6^ substitutions/site/year (40). Since Segawa et al. (68) considered only a slowly evolving, highly conserved gene, the 16S rRNA gene, in their mutation rate calculations, and most of the genome mutation rates for bacteria analyzed by Gibson and Eyre-Walker fell between 1 × 10^−7^ and 1 × 10^−6^ substitutions/site/year, this range likely captures the accumulation rates observed in *Microcystis* present in western Lake Erie. However, since this rate has not yet been determined for *Microcystis*, we consider a hypermutation rate observed in a few bacteria that is on the order of 1 × 10^−4^ substitutions/site/year (40). Therefore, in order to consider a wide range of mutation accumulation rates, we report the time of divergence for the range of 1 × 10^−7^ to 1 × 10^−4^ substitutions/site/year. The time of divergence was completed for each pairwise comparison between samples for the whole genome as well as the *mcyB* and *mcyC* genes.

### Figure creation and statistical analysis.

All statistical analyses and plots were created using R and R Studio 3.5.1 (69). The R packages ggplot2 (70), ggthemes (71), gridExtra (72), viridis (73), and ggpubr (74) aided in figure creation. Adobe Illustrator v25.1 (75) was used to generate schematic figure drawings. Maps were rendered in QGIS using the Quick Map Services plug-in (76). Metadata can be found in Table S3.

### Data availability.

Sequences have been deposited in the NCBI database under the BioProject accession number PRJNA702128. Metagenomic and metatranscriptomic raw reads can be found in the NCBI database under the BioProject accession numbers PRJNA464361 and PRJNA370007, respectively.
