# Supplementary material for: Metagenomic and Metatranscriptomic Insights into Population Diversity of Microcystis Blooms: Spatial and Temporal Dynamics of mcy Genotypes, Including a Partial Operon That Can Be Abundant and Expressed
Source: Appl Environ Microbiol. 2022 Apr 19;88(9):e02464-21. doi: 10.1128/aem.02464-21 (PMC9088275; doi:10.1128/aem.02464-21)
Supplement: Supplemental file 1 — Tables S1 to S4. Download aem.02464-21-s0001.pdf, PDF file, 0.2 MB [file aem.02464-21-s0001.pdf]

Supplemental Tables

Table S1: Pearson's correlations for mcy genotype or mcy B1/C1 and all environmental variables tested.

**Operon Status**	**Environmental Variable**	**R value**	**P-value**
Complete	pH	0.513	0.05
Partial	pH	-0.0318	0.91
Absent	pH	-0.541	0.037
Complete	NO3	0.621	0.013
Partial	NO3	-0.525	0.044
Absent	NO3	-0.359	0.19
Complete	NH4	-0.082	0.77
Partial	NH4	-0.52	0.048
Absent	NH4	0.403	0.14
Complete	Temp	0.467	0.09
Partial	Temp	-0.288	0.31
Absent	Temp	-0.336	0.24
Complete	Distance from Maumee	-0.0582	0.84
Partial	Distance from Maumee	0.0253	0.92
Absent	Distance from Maumee	0.0482	0.86
Complete	Particulate MC	0.338	0.3
Partial	Particulate MC	-0.358	0.28
Absent	Particulate MC	-0.149	0.66
Complete	Phycocyanin	0.051	0.86
Partial	Phycocyanin	0.461	0.076
Absent	Phycocyanin	-0.342	0.21
Complete	chlA	0.19	0.49
Partial	chlA	0.267	0.33
Absent	chlA	-0.369	0.17

Complete	SRP	-0.432	0.11
Partial	SRP	0.542	0.04
Absent	SRP	0.142	0.61
Complete	TP	-0.242	0.21
Partial	TP	0.371	0.17
Absent	TP	0.149	0.5963
Complete	DOC	0.0251	0.93
Partial	DOC	0.479	0.07
Absent	DOC	-0.317	0.25
C1/B1	pH	0.156	0.57
C1/B1	NO3	-0.189	0.5
C1/B1	NH4	-0.149	0.59
C1/B1	Temp	0.222	0.44
C1/B1	Distance from Maumee	0.373	0.17
C1/B1	Particulate MC	-0.157	0.65
C1/B1	Phycocyanin	-0.119	0.67
C1/B1	chlA	-0.12	0.67
C1/B1	SRP	-0.175	0.53
C1/B1	TP	-0.258	0.35
C1/B1	DOC	0.58	0.022

Table S2: Pairwise comparison conANI and and popANI scores between samples from the 2014 cyanoHAB

Sample 1	Sample 2	conANI	popANI
49637	53598	0.975853	0.993557
53598	53602	0.980335	0.993974
53598	53603	0.980104	0.994138
49636	53598	0.975031	0.994559
53598	53601	0.982174	0.996385
49629	53598	0.980406	0.996732
49625	53598	0.980632	0.997218
49633	53598	0.982437	0.99726
42896	49636	0.973014	0.998112
49636	53603	0.973117	0.998277
49615	53603	0.976791	0.998323
49636	53602	0.973666	0.998368

49637	53599	0.979177	0.998376
49615	49636	0.973564	0.998404
49615	53602	0.977715	0.998477
49636	53599	0.977736	0.998536
49637	53603	0.975888	0.998571
49615	49637	0.975224	0.998604
42896	49637	0.97495	0.998692
49637	53602	0.976254	0.99871
49636	53601	0.975956	0.99871
53599	53602	0.983109	0.998864
49636	53600	0.97606	0.998898
49637	53601	0.977883	0.998918
53599	53603	0.982578	0.99892
49622	49636	0.977663	0.998935
49633	49637	0.978624	0.998995
49622	49637	0.979395	0.999009
49629	49636	0.973641	0.999012
53598	53599	0.98553	0.999059
49637	53600	0.978466	0.999084
49633	49636	0.976729	0.999134
49622	53598	0.986119	0.99917
49625	49636	0.973734	0.999173
42896	53603	0.977672	0.999184
49629	49637	0.976539	0.999356
42896	53602	0.978631	0.999406
49625	49637	0.976671	0.999412
49615	49629	0.978125	0.99944
49629	53599	0.983057	0.999451
49615	53601	0.982168	0.999502
53599	53601	0.986969	0.999521
53600	53603	0.992324	0.999548
49615	49633	0.981238	0.999549
53601	53603	0.991818	0.999585
49625	53599	0.98347	0.99959
49633	53599	0.987418	0.999594
49622	53603	0.988381	0.999597
53601	53602	0.992195	0.9996
42896	53599	0.983636	0.999624
42896	49633	0.981637	0.999631
49633	53603	0.991151	0.999641
49615	49625	0.979387	0.999642
42896	49629	0.978813	0.999654
53599	53600	0.98705	0.999659
42896	53598	0.982104	0.999664

49622	53602	0.98901	0.999677
49633	53602	0.991732	0.99969
53598	53600	0.985256	0.999705
42896	49625	0.97972	0.999715
49633	53600	0.991518	0.999719
53600	53601	0.992117	0.999721
49622	53599	0.989323	0.999725
49629	53601	0.991937	0.999727
53600	53602	0.993341	0.999732
42896	53601	0.982676	0.999733
49615	53599	0.984306	0.999791
49625	53601	0.992151	0.999794
49622	49633	0.990613	0.999794
49629	53600	0.992646	0.999801
49615	53600	0.983333	0.999802
49622	53601	0.990574	0.999805
42896	53600	0.983615	0.999827
49629	49633	0.991593	0.999842
49633	53601	0.992529	0.999842
49615	53598	0.983228	0.999845
49625	53600	0.993089	0.999847
49622	49629	0.989099	0.99986
49622	53600	0.991761	0.999872
49625	53603	0.996214	0.999876
53602	53603	0.997936	0.999877
49625	49633	0.991688	0.999883
49629	53603	0.997068	0.999892
42896	49615	0.9955	0.99992
49622	49625	0.989721	0.99992
49629	53602	0.997882	0.999952
49615	49622	0.987082	0.999955

Table S3: Paired environmental parameters for 2014 cyanoHAB

Station	Date	NO3 (mg/L)	NH4 (ug/L)	Temperature (°C)	Latitude	Longitidue	Distance to Maumee River (km)	Shore	pH	Particulate _Microcystins (ug/L)	Phycocyanin (ug/L)	CHLa (ug/L)	SRP (ug/L)	TP (ug/L)	DOC (um)
WE12	8-Jul-14	0.34	16.16	23.5	NA	NA	17.44	Near	8.33	NA	0.12	6.98	0.2	34	214
WE12	4-Aug-14	0.62	3.25	24.6	41 42.157	83 15.781	16.35	Near	9.29	9.28	45.43	54.46	0.1	44.4	384
WE2	4-Aug-14	0.56	3.03	23.1	41 45.912	83 19.835	12.3	Near	9.2	4.33	39.01	29.44	1.45	32	462
WE4	4-Aug-14	0.14	1.64	24.6	41 49.714	83 11.654	25.64	Off	9.02	1.54	15.38	18.11	0.55	22.1	332
WE2	29-Sep-14	0.15	0.1	19.4	41 45.884	83 20.107	11.95	Near	8.84	4.2	205.5	66.63	21.22	65.3	601
WE12	29-Sep-14	0.14	0.44	19.5	41 42.185	83 15.546	16.7	Near	8.74	0.6	21.87	22.25	7.98	69.2	398
WE4	29-Sep-14	0.19	0.33	19.9	41 49.608	83 11.684	25.44	Off	8.58	0.2	3.65	9.21	0.1	17.7	464
WE2	20-Oct-14	0.16	6.57	12.5	41 45.827	83 19.855	12.2	Near	8.04	NA	2.12	7.23	9.16	44.3	287
WE12	20-Oct-14	0.16	5.18	13	41 42.269	83 15.663	16.54	Near	7.98	NA	0.54	5	13.27	45.2	419
WE2	21-Jul-14	0.52	2.93	23.5	41 45.873	83 19.849	12.23	Near	8.38	4.94	5.13	16.26	0.1	32.1	311
WE4	29-Jul-14	0.31	9.39	22.6	41 49.637	83 11.713	25.53	Off	8.59	1.01	4.77	6.12	1.01	16.5	328
WE12	25-Aug-14	0.03	2.58	25	41 42.298	83 15.377	16.88	Near	9.29	3.1	45.72	38.28	3.68	98.1	415
WE4	8-Sep-14	0.07	5.28	NA	41 49.585	83 11.629	25.55	Off	8.86	0.8	15.81	26.24	0.27	30.6	328
WE12	23-Sep-14	0.15	3.14	17.9	41 42.196	83 15.499	16.72	Near	8.33	0.9	17.55	16.97	17.39	60.3	495
WE2	6-Oct-14	0.15	11.18	15.3	41 45.795	83 19.942	12.08	Near	8.04	NA	6.24	8.06	12.65	53	502

Table S4: Gene lengths used for read normalization. Lengths were determined to be the average of all *mcy* genes used in mapping databases.\

Gene	Length (bp)
mcyJ	1009.5
mcyI	931.5
mcyH	1747.75
mcyG	7896.947
mcyF	755.5714
mcyE	10456.26
mcyD	11651.53
mcyA	8439.632
mcyB	6382.059
mcyC	3861.6
mcyB1	955
16s	253
